# Comparing Climate Change and Species Invasions as Drivers of Coldwater Fish Population Extirpations

**DOI:** 10.1371/journal.pone.0022906

**Published:** 2011-08-10

**Authors:** Sapna Sharma, M. Jake Vander Zanden, John J. Magnuson, John Lyons

**Affiliations:** 1 Center for Limnology, University of Wisconsin-Madison, Madison, Wisconsin, United States of America; 2 Wisconsin Department of Natural Resources, Madison, Wisconsin, United States of America; Institute of Marine Research, Norway

## Abstract

Species are influenced by multiple environmental stressors acting simultaneously. Our objective was to compare the expected effects of climate change and invasion of non-indigenous rainbow smelt (*Osmerus mordax)* on cisco (*Coregonus artedii*) population extirpations at a regional level. We assembled a database of over 13,000 lakes in Wisconsin, USA, summarising fish occurrence, lake morphology, water chemistry, and climate. We used A1, A2, and B1 scenarios from the Intergovernmental Panel on Climate Change (IPCC) of future temperature conditions for 15 general circulation models in 2046–2065 and 2081–2100 totalling 78 projections. Logistic regression indicated that cisco tended to occur in cooler, larger, and deeper lakes. Depending upon the amount of warming, 25–70% of cisco populations are predicted to be extirpated by 2100. In addition, cisco are influenced by the invasion of rainbow smelt, which prey on young cisco. Projecting current estimates of rainbow smelt spread and impact into the future will result in the extirpation of about 1% of cisco populations by 2100 in Wisconsin. Overall, the effect of climate change is expected to overshadow that of species invasion as a driver of coldwater fish population extirpations. Our results highlight the potentially dominant role of climate change as a driver of biotic change.

## Introduction

Global biodiversity is threatened by environmental stressors such as climate change, habitat loss, and biological invasions [1]. Forecasting the effects of environmental stressors has received attention in recent years and suggests that a single environmental stressor can lead to the local extirpation of native species (e.g., [2]–[4]). Climate change and biological invasions are two of the foremost threats to aquatic ecosystems [1], [3], [5]. Global climatic change is expected to alter species distributions, community composition, and ecosystem structure [6]–[7]. Climate change will have profound impacts on thermal habitat, distribution, and growth of freshwater organisms [8]–[11]. Under climate change scenarios, coldwater fishes may lose suitable thermal habitat in the south, but may also expand their range northward, and warmwater fish species may expand their range [8], [12]–[13]. The invasion and the northward range expansion of non-indigenous species may have serious consequences for native species [11], [14], [16] as the invasion of non-indigenous species can have large ecological [15]–[18] and economic impacts [19].

Coldwater fishes, such as cisco [*Corgeonus artedii*] require cold water temperatures, high dissolved oxygen concentrations, and oligotrophic conditions, and thereby are sensitive indicators of environmental change [20]. In Wisconsin, cisco are close to the southern edge of their range and are listed as a species of special concern [21]–[22]. Cisco live in larger and deeper inland lakes with cold, well-oxygenated deep waters [21], [23]–[24]. Under climate change scenarios, as air temperatures increase, epilimnion and hypolimnion water temperatures are expected to increase [8], [25]–[26]. As water temperatures increase, the duration of the lake stratification period is expected to increase, isolating the deep waters from exchanges with the atmosphere, making it more likely that metabolic activity will reduce dissolved oxygen concentrations in the hypolimnion to stressful or lethal levels [27]–[29]. The combination of warmer water temperatures and lower dissolved oxygen concentrations under climate change scenarios in larger, deeper lakes typically suitable for coldwater fishes could result in their extirpation.

Cisco are sensitive to the introduction of non-native rainbow smelt (*Osmerus mordax*). Rainbow smelt is native to the northeastern coast of North America and was introduced to the Laurentian Great Lakes in the 1920s [30]. In Wisconsin, rainbow smelt have been introduced into lakes deliberately by anglers for sport fishing purposes [31]–[32]. Furthermore, fertilized eggs of rainbow smelt may have been unintentionally introduced into lakes by residents cleaning smelt on their piers [31]. When rainbow smelt invade a system, they negatively interact with native species through predation and competition [30]. Invasion of rainbow smelt has been linked directly to changes in zooplankton community composition [33], decline in recruitment of walleye (*Sander vitreus*) [34], and extirpation of cisco and yellow perch (*Perca flavescens*) [35]. For example in Sparkling Lake, Wisconsin, the cisco population was extirpated through predation-induced recruitment within eight years of detection of rainbow smelt [32], [35]–[36].

Our overall goal was to predict the effects of climate change and biological invasions on local extirpations of cisco, a coldwater fish species. Specific objectives were three-fold: First, to predict the impact of climate change and the underlying uncertainty in climate change scenarios on cisco extirpations; Second, to predict the impacts of rainbow smelt invasion on cisco extirpations, and lastly, to compare the relative importance of climate change and invasion of rainbow smelt on cisco extirpations. We incorporated uncertainties to our projections using a framework that could be expanded to include additional stressors.

## Methods

### Ethics Statement

The current status of cisco was further documented through extensive field research and personal communication with Wisconsin Department of Natural Resources staff by John Lyons. As a biologist for the state of Wisconsin, John Lyons has the necessary authority and permission to survey and collect all species of fish from all waters of the state.

### Data acquisition

Geo-referenced lake-specific data were collected for 13,052 lakes in Wisconsin from a variety of sources including the North Temperate Lakes Long Term Ecological Research (NTL-LTER) program, Wisconsin GAP (Geographic Approach to Planning for Biological Diversity) database, Wisconsin Department of Natural Resources databases, refereed publications, government reports, and dissertations. From the aforementioned databases, a suite of variables describing lake morphology, water chemistry, physical habitat, and fish species occurrence were compiled. Environmental variables retained in the final dataset were: surface area (hectares), maximum depth (metres), perimeter (kilometres), Secchi depth (metres), pH, conductivity (µS/cm), and mean annual air temperatures (°C). For water chemistry variables, annual averages were used in the dataset.

**Figure 1:**
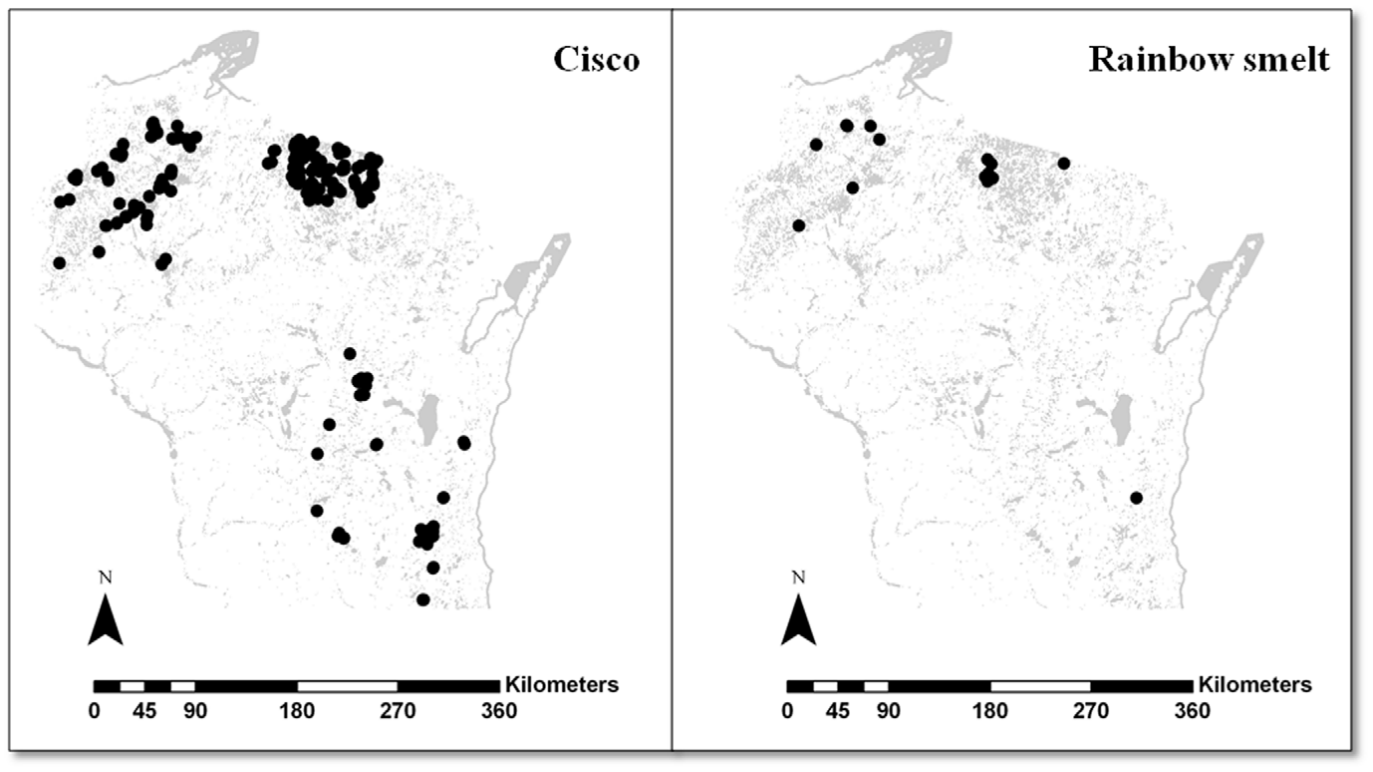
Species occurrence in Wisconsin. Current occurrence of a) cisco and b) rainbow smelt in Wisconsin lakes. Currently, cisco are present in 184 lakes and rainbow smelt occur in 24 lakes.

Wisconsin has 184 lakes with confirmed cisco occurrences, found mostly in northern Wisconsin (Figure 1). The current status of cisco was further documented through extensive field research and personal communication with the staff of the Wisconsin Department of Natural Resources. Currently, 24 lakes have rainbow smelt. Mercado-Silva et al. (2006) estimated that 578 lakes are suitable for rainbow smelt in Wisconsin based on classification tree models [37].

The final dataset used to develop and validate the models consisted of 4402 lakes. The dataset was first reduced based on availability of environmental variables at a broad geographic scale. The data were divided randomly into a training and a validation dataset with the same large-scale geographic coverage in both datasets. Seventy-five percent of the data comprised the training dataset and were used to develop the statistical models. Twenty-five percent of the data was retained for the validation dataset to test the performance of the models. The training dataset consisted of 3302 lakes with cisco present in 139 lakes. The independent, validation data was comprised of 1100 lakes with cisco present in 45 lakes (Table 1).

**Table 1:** Datasets used to develop and validate cisco occurrence models.

Dataset	No. of lakes	Cisco occurrence
Total	4402	184
Training	3302	139
Validation	1100	45

Number of lakes and occurrence of cisco in the total, training, and validation datasets used to develop and validate the cisco occurrence models.

Current air temperatures and scenarios of future mean annual air temperatures were obtained from the Wisconsin Initiative on Climate Change Impacts (WICCI) Climate Working Group [38]. Mean annual air temperatures were statistically downscaled for Wisconsin on a 0.1° latitude ×0.1° longitude grid. Climate data were summarised for three time periods: 1961–2000, 2046–2065, and 2081–2100 and averaged over these three sets of years as suggested by the Intergovernmental Panel on Climate Change (IPCC) to reduce temporal variation in climate. Then, projected air temperatures from 15 general circulation models and the IPCCs A1, A2 and B1 scenarios (although not all general circulation models incorporated all three scenarios) totalling 78 climate change scenarios were used to develop future projections of cisco occurrence [42; Table S1]. The A1, A2 and B1 scenarios incorporate a range of variation in greenhouse gas emissions inferred for various time periods in the 21^st^ century. The A1 scenario is the most extreme and assumes the highest greenhouse gas concentrations, followed by the A2 and B1 scenarios [39].

### Data analyses

#### i. Current cisco distribution

Multicollinearity among variables was evaluated using bivariate plots and correlation analyses to determine which variables with broad geographic coverage should be retained prior to regression analyses. Variables were log transformed, as necessary, to satisfy assumptions of normality-andresidual plots were examined to identify whether assumptions of homoscedasticity were satisfied. Environmental variables retained in the final dataset were: surface area; maximum depth, perimeter, Secchi depth, pH, conductivity, and mean annual air temperatures. Data on each of these variables was present in the final dataset (n = 4402 lakes). Mean annual air temperatures were summarised as the average of 1961–2000 air temperatures as suggested by the IPCC and annual averages of water chemistry variables were included in the dataset. Variables entering the model were selected using a forward selection procedure to select variables that were significant predictors of cisco occurrence [40].

Multiple logistic regression models were constructed for Wisconsin lakes to evaluate the relationship between cisco occurrence and physical habitat, water chemistry, and climatic predictor variables. In a logistic regression, response variables are subject to a logit transformation, whereas predictor variables are based on a linear combination using maximum likelihood [9], [41]. Significance values were set at a value of α = 0.05 for predictor variables to enter and remain in the model. Logistic regression models were tested on the validation dataset comprised of 1100 lakes to determine how well the models predicted cisco presence and absence on a dataset that was not used to develop the models.

Instead of designating cisco presence by the traditional decision threshold of 0.50, we constructed Receiver Operating Characteristic (ROC) curves to identify the threshold that would maximise sensitivity and specificity [41]–[42]. ROC curves are particularly useful with datasets that have unequal species presence and absence as logistic regression tends to produce scores that are biased towards the more prevalent group [42]. ROC analyses were designed to identify thresholds that would predict sensitivity [correctly predicting species presence] and specificity [correctly predicting species absence] equally well. Based on the ROC analyses and the optimal decision threshold, we constructed “confusion matrices” using the independent, validation datasets [42]. The confusion matrix summarises true absence, true presence, false absence, and false presence, which can then be used to calculate the overall classification rate, sensitivity, specificity, and Cohen's Kappa statistic [41]–[42]. Cohen's Kappa statistic determines how well the model performed in its ability to correctly predict species presence or absence after accounting for chance effects. As such, Cohen's Kappa statistic provides an indication of whether the model performed differently than expected by chance [41]–[42]. Kappa values greater than 0.4 indicate good model performance. The combination of good overall classification rates, sensitivity, specificity, and Kappa values, increased our confidence in using the model to examine responses to the different climate change scenarios [8]–[9]. All data manipulation and statistical analyses were performed in the R-language environment [43].

#### ii. Extirpation of cisco under climate change scenarios

The percent of cisco populations extirpated was summarised as a probability density function of cisco loss versus average change in mean annual air temperature based on each of the 78 climate change scenarios. Cisco were expected to be extirpated within a lake if the log likelihood of cisco occurrence was below the threshold of species presence designated by the ROC analysis. Percentages of cisco extirpations were summarised as the percent of cisco extirpated in the 184 lakes in each climate change scenario.

#### iii. Extirpation of cisco under rainbow smelt invasion

Two major filters were calculated and assumed to contribute to the extirpation of cisco under rainbow smelt invasion: i) the probability of dispersal of rainbow smelt to a lake containing cisco (P(D)) and ii) the probability of a cisco extirpation owing to the invasion of rainbow smelt (P(E)).

The first filter contributing to the extirpation of cisco under rainbow smelt invasion is the ability of rainbow smelt to disperse to lakes currently containing cisco (P(D)). The probability of rainbow smelt dispersal was determined as follows:

- Based on matching the current distribution of cisco (184 lakes) and potential future distribution of rainbow smelt (578 lakes) in Wisconsin [37], cisco presently occur in 125 lakes that are identified as being potentially suitable for rainbow smelt. (*Nc* = 125; *Ns = *578);
- The dispersal of rainbow smelt depends upon the invasion rate. The observed invasion rate of rainbow smelt in Wisconsin is approximately 0.5 new invasions/year [37]. We incorporated uncertainty into invasion rate of rainbow smelt by using scenarios of 20, 50, 100, 200, and 500% the observed invasion rate (*Inv*);
- Calculate the number of years required for rainbow smelt to invade the 578 suitable lakes in Wisconsin (*t(s)*);
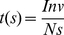
(1)
- Calculate the number of lakes invaded by rainbow smelt in 100 years. 100 years corresponds to the time scale of the climate change scenarios (*t(c)*);
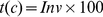
(2)
- Therefore, the probability of dispersal of rainbow smelt to lakes containing cisco (P(D)) is
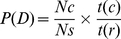
(3)

The second filter contributing to the extirpation of cisco under rainbow smelt invasion is the probability of cisco extirpation owing to the invasion of rainbow smelt (P(E)). Based on a literature review, we estimated that rainbow smelt cause cisco extirpations approximately 20% of the time [35]. We incorporated uncertainty into the impact rate of rainbow smelt on cisco extirpations by using scenarios of 20, 50, 100, 200, and 500% the estimated extirpation rate.

Combining the two filters to calculate the probability of cisco extirpations from rainbow smelt invasion (P(S)) resulted in an estimate of the fraction of cisco extirpated owing to the ability of rainbow smelt to invade a cisco lake, and if invaded, the ability of rainbow smelt to extirpate cisco. The probability of cisco extirpations from rainbow smelt invasion was calculated as follows:
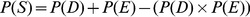
(4)


#### iv. Extirpation of cisco under rainbow smelt invasion and climate change scenarios

Percent cisco extirpation was summarised as the percentage of cisco extirpated in each combination of climate change and rainbow smelt invasion scenarios. Assuming that the impact of climate change and rainbow smelt invasion are independent on cisco extirpations, to estimate the extirpation of cisco (P(E)), the probability of extirpation of cisco by 2100 under climate change scenarios (P(C)) was combined with the probability of extirpation of cisco by 2100 under rainbow smelt invasion (P(S)), such that:
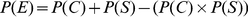
(5)


## Results

### i. Current cisco distribution

Cisco occurrence follows a logit transformation and was best predicted by:
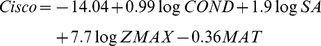
(6)
*where* COND  =  conductivity, SA  =  surface area, ZMAX  =  maximum depth, and MAT  =  mean annual air temperature. In general, cisco tended to be found in larger, deeper lakes in colder regions of the state and in lakes with higher conductivity values. The sample size used to construct the model was 3294 lakes and the model had an Akaike Information Criterion (AIC) value of 528.3. The AIC value was the lowest of all significant models using the various combinations of variables, thereby indicating that this model was the most parsimonious (Table 2). The deviance of the model was 52.1%.

**Table 2:** Logistic regression models predicting cisco occurrence in Wisconsin.

Model	Predictor variables	P-value	AIC
1	Zmax + SA + Cond - mat	0.02	528.3
2	Zmax + SA	<0.001	530.6
3	Zmax + SA + Secchi + Cond	0.28	532.2
4	Zmax + SA + Cond + Secchi - mat	0.33	529.4
5	Zmax + SA + Cond	0.38	531.8
where	Zmax = maximum depth		
	SA = surface area		
	Cond = conductivity		
	mat = mean annual air temperature		
	Secchi = Secchi depth		

Logistic regression models predicting cisco occurrence in Wisconsin lakes providing an indication of significance (P-value) and the Akaike Information Criterion (AIC) value. The significant model with the lowest AIC value (Model 1) is the best model for predicting cisco occurrence in Wisconsin lakes.

The cisco occurrence model was evaluated on an independent, validation dataset of 1096 lakes. The overall classification success of the cisco occurrence model was approximately 93%. Eighty-three percent of cisco presence (sensitivity) and 93% of cisco absence (specificity) were correctly predicted by the model. Cohen's Kappa statistic was 0.43 suggesting that model performance is significantly better than expected by chance.

### ii. Extirpation of cisco under climate change scenarios

**Figure 2:**
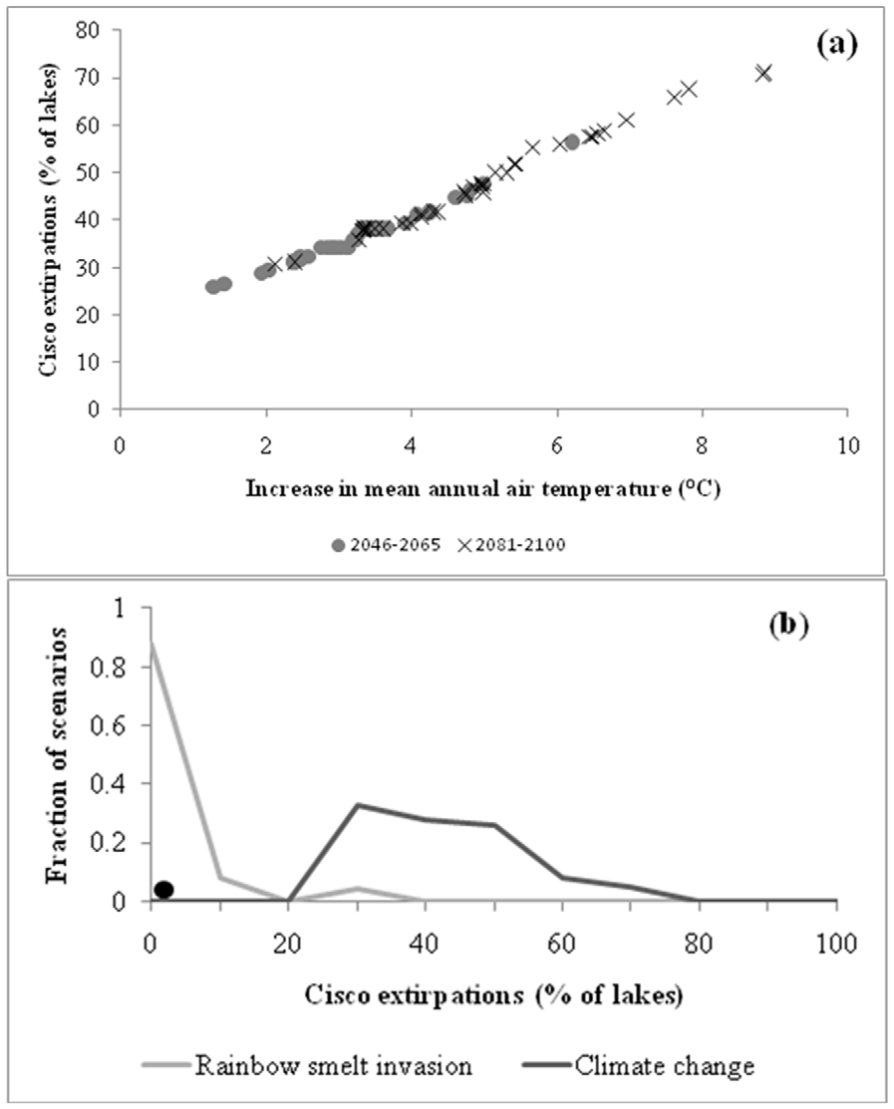
Impacts of climate change on cisco extirpations. a) Percent cisco extirpation with increases in average mean annual air temperature (°C) resulting from climate change across Wisconsin lakes. Percent cisco extirpation was predicted by incorporating estimates of changes in mean annual air temperature (°C) derived from the 78 climate change scenarios into the cisco occurrence model (Equation 6). Predicted cisco extirpations for mid-century (2046–2065) are depicted by grey circles and predicted cisco extirpations for late century are depicted by black crosses. b) Fraction of scenarios predicting cisco extirpations (a percentage of current cisco populations) in Wisconsin resulting from climate change versus the invasion of rainbow smelt. Cisco extirpations from climate change incorporated uncertainty from 78 climate change scenarios. Cisco extirpations from rainbow smelt invasion incorporated uncertainty on rainbow smelt invasion rates and the direct extirpation of cisco as a result of rainbow smelt invasion. The solid circle represents the current best estimate of cisco extirpations under rainbow smelt invasion (assuming a spread rate of 0.5 and an impact rate of 0.2).



Cisco extirpations are expected to increase with mean annual air temperature (Figure 2a). The probability density function suggested that the relationship between percent cisco loss (%) and increases in mean annual air temperatures (°C) can be summarised as follows:

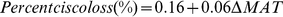
(7)
*where* ΔMAT  =  average changes in mean annual air temperature (°C) from 1961–2000 to 2046–2065 or 2081–2100 across Wisconsin lakes.

We found, not surprisingly, that the largest percent cisco extirpations were expected with the A2 climate change scenarios (business-as-usual) and the best-case climate change scenario (B1) predicted the lowest loss of cisco both for the middle and end of the century (Table S1).

### iii. Extirpation of cisco under rainbow smelt invasion

Cisco occur in 125 lakes that are suitable for rainbow smelt. The risk of cisco extirpation depends upon the invasion and impact rate of rainbow smelt. The uncertainty analysis revealed that most rainbow smelt invasion scenarios projected low percentages of cisco extirpations (Table 3; Figure 2b). The best estimate for rainbow smelt invasion predicted extirpations of 1.2% of cisco populations by 2100, assuming a smelt invasion rate of 0.5 lakes/year [37] and an impact rate of 20% ([35]; Table 2].

**Table 3:** Cisco extirpation (percent of current cisco populations) across a range of rainbow smelt invasion scenarios.

			Invasion rate		
Impact rate	0.1	0.25	0.5	1	2.5
**0.04**	0.05	0.1	0.2	0.5	1.2
**0.1**	0.1	0.3	0.6	1.2	3.1
**0.2**	0.2	0.9	***1.2***	2.5	6.2
**0.4**	0.5	1.5	2.5	4.9	12.4
**1**	1.2	3.1	6.2	12.4	30.9

Cisco extirpations resulting from rainbow smelt invasion, using a range of rainbow smelt invasion and impact scenarios. The most likely scenario is an invasion rate of 0.5 lakes/year (the observed invasion rate of rainbow smelt in Wisconsin [37]) and an impact rate of 20% (based on a literature review [35]) is italicized and bold.

### iv. Extirpation of cisco under rainbow smelt invasion and climate change scenarios

Assuming that the risk of cisco extirpation from climate change and rainbow smelt invasion are independent, the overall risk of cisco extirpation from climate change greatly exceeds that of rainbow smelt invasion (Figure 2b). Scenarios combining the effects of climate change and rainbow smelt invasion reveal the strong temperature dependence of cisco extirpations. For a given warming scenario, rainbow smelt invasion scenarios have relatively little effect on cisco extirpation (Table 4).

**Table 4:** Predicted cisco extirpations from a series of climate change and rainbow smelt invasion scenarios.

			Invasion rate	
Impact rate		0.25	0.5	1
*a) 2°C increase*				
	**0.1**	31	31	31
	**0.2**	31	31	32
	**0.4**	32	32	34
*b) 4°C increase*				
	**0.1**	40	40	40
	**0.2**	40	40	41
	**0.4**	40	41	42
*c) 6°C increase*				
	**0.1**	56	56	56
	**0.2**	56	56	57
	**0.4**	57	57	58
*d) 7.8°C increase*				
	**0.1**	68	68	68
	**0.2**	68	68	69
	**0.4**	68	69	69

Predicted cisco extirpations (percent of lakes) across a range of climate change and rainbow smelt invasion scenarios.

## Discussion

We predicted the effects of two environmental stressors acting on the local extirpation of a native species. We used cisco as a sentinel of climate change and biological invasions, as the species requires high quality habitat and is a species of special concern in Wisconsin [22].

### Current cisco distribution

Wisconsin has 184 lakes with confirmed historical cisco occurrences, with the majority of these in the north. Five lakes in Wisconsin have reduced cisco populations, whereby their abundances have declined substantially in recent years. These cases have been attributed to either eutrophication or the invasion of rainbow smelt, or both. Six cisco populations have been extirpated in recent years, as cisco have not been found despite repeated targeted sampling. Cisco extirpations have been attributed to the loss of suitable habitat as water temperatures warmed with the associated decline in summer hypolimnetic oxygen concentrations, and because of predation from rainbow smelt, walleye, and muskellunge (*Esox masquinongy*; [22]).

Cisco tend to be found in larger, deeper, cooler lakes with high conductivity. In Wisconsin, conductivity is an indicator of lake type, such that larger, non-dystrophic lakes have higher conductivity than smaller, dystrophic lakes. During stratification, cisco venture into the warmer epilimnion if the hypolimnion is depleted of oxygen [44]. The late summer period is especially critical in some lakes as epilimnetic water temperatures can become too warm and hypolimnetic oxygen concentrations can become too low [45] potentially leading to decreased growth rates [46] and summerkills of cisco [21]. Hypolimnetic oxygen concentrations would be expected to decline more in eutrophic than oligotrophic lakes and present a greater threat to cisco owing to the loss of cold oxygenated refuge areas [25], [29], [47]. For example, in Halfmoon Lake, Indiana, summer kills of cisco, particularly of larger and older individuals, were common owing to high water temperatures and low dissolved oxygen concentrations [48]. Adult cisco are more sensitive to warmer temperatures than are young-of-year cisco, which are more tolerant of higher temperatures and low dissolved oxygen concentrations [49].

### Extirpation of cisco under climate change scenarios

The number of cisco extirpations depends on the magnitude of climate warming. For example, an increase in mean annual air temperatures across Wisconsin of 5°C is expected to result in a loss of 50% of cisco populations. Our application of the conservative MIROC-A1 climate change scenario developed by the Division of Climate System Research in Tokyo, Japan, projects the extirpation of over 70% of cisco populations in Wisconsin by the end of the century. The use of multiple general circulation models has been implemented only recently in ecological studies (e.g., [39], [50]), and previous studies did not incorporate the full array of available climate change models and scenarios. Incorporating uncertainty into climate change projections by employing a suite of climate change models, scenarios, and time-frames, reflects the uncertainty of climate change scenarios and increases our ability to project the potential range of impacts of climate change on native species [39]. The uncertainty in climate change projections arises from the large variation in inferred changes in climate conditions for each general circulation model and corresponding scenarios [51]. The variation in projections results from the difference in each general circulation model in its initial assumptions, the variability in physical processes, the types of climate feedback mechanisms, and the spatial resolution at which these processes are acting [51]–[52].

Coldwater fishes require not only cold temperatures in the deep waters, but also sufficient dissolved oxygen. In stratified lakes, juvenile and adult cisco inhabit the hypolimnion during the summer where cooler water temperatures and often higher dissolved oxygen concentrations prevail [22]–[23]. Global mean air temperatures are predicted to increase by between 1.4 and 5.8°C by the year 2100, with variability among estimates resulting from different expectations about economic development and population growth and assumptions in the General Circulation Models used to forecast temperatures [51]. As air temperatures increase, epilimnetic water temperatures are expected to increase [8], [26]. Warmer epilimnetic water temperatures may lead to a larger thermal gradient, a shallower thermocline [53], and warmer hypolimnetic temperatures [25], [54]. Under climate change scenarios, the intensity of stratification is expected to increase which may reduce the amount of coldwater habitat available during the summer [55]. For example, hypolimnetic water temperatures in deep Minnesota lakes are projected to increase by 1°C in the north and by 3°C in the south [25]. This would still be in the range suitable for cisco. The primary mechanistic relationship between air temperatures and the hypolimnion of lakes in large, deep lakes typical for coldwater fishes, is that thermal stratification would start sooner and end later resulting in greater hypolimnetic oxygen depletion over the longer period of stratification. Warmer hypolimnetic temperatures are also expected. These two factors lower dissolved oxygen and warmer hypolimnetic temperatures physiologically stress coldwater fishes [29], [56]–[57].

Climate change in conjunction with changes in lake productivity owing to increased runoff from extreme precipitation events would be expected to exacerbate lake eutrophication and present a greater threat to cisco. Climate change is expected to lead to warmer air and epilimnetic water temperatures [8] and a longer growing season [6] which could lead to higher pelagic primary productivity [58]. Increased primary productivity will lead to greater respiration ultimately leading to increased depletion rates of dissolved oxygen in the hypolimnion which can approach stressful or lethal levels for coldwater fishes [56], [59]–[60]. Shallow, eutrophic lakes that are deep enough to have deep, cold waters are expected to be at a higher risk [57]. In conjunction with increases in air temperature, more extreme rainfall events attributed to climate change [61] could lead to greater runoff, increased nutrient inputs, higher primary productivity, and consequently a decline in coldwater fish habitat suitability [56]. As lake productivity is partly governed by the input of nutrients from the watershed, a reduction in nutrient inputs and improved land-use management could help decrease the likelihood of habitat degradation for coldwater fishes [45]–[46].

### Extirpation of cisco under rainbow smelt invasion

Cisco populations are further under threat of extirpation from the invasion of rainbow smelt. Rainbow smelt are native to the Atlantic coast of north-eastern North America, and have invaded the Laurentian Great Lakes and surrounding inland lakes [30]. Rainbow smelt invasion and impact rates were deemed to be the most important factors in governing the probability of cisco extirpation due to rainbow smelt invasion. As a result of the uncertain rates of these two factors, a suite of rainbow smelt dispersal and impact scenarios were investigated. Generally, most scenarios of rainbow smelt invasion did not result in many cisco extirpations. For example, the best estimate of present-day rainbow smelt spread and impact rates predicts the extirpation of only 1.2% cisco populations by 2100.

Currently, the estimated invasion rate in Wisconsin lakes is 0.5 lakes per year [37], [62]. Several dispersal vectors may be contributing to the spread of rainbow smelt in invaded regions. Urban development exhibits a strong relationship with rainbow smelt occurrence, suggesting a strong role of human-mediated dispersal relative to natural dispersal in some lakes [30], [62]. The invasion rate may increase or decrease depending upon the degree of human-mediated dispersal, the rate of natural dispersal via connecting waterways, the proximity of invaded lakes to uninvaded lakes, and management of invasive species movement and establishment [31], [37], [62].

The impact of rainbow smelt invasion on cisco varies among lakes, depending on lake characteristics and timing of invasion. For example, in Sparkling Lake, Wisconsin, rainbow smelt were first detected in 1982, and were found to overlap spatially in the hypolimnion and share a similar thermal niche with cisco [35]. Within five years, cisco declined to low abundances [63]. Adult rainbow smelt prevented recruitment by preying on the larvae of cisco within eight years after first detection of rainbow smelt, cisco were extirpated [35]. In contrast, in Ontario lakes, 26% of cisco populations experienced lower abundances following rainbow smelt invasion [30]. In Michigan, 14% of all cisco populations were extirpated which was attributed to habitat deterioration or invasion of rainbow smelt and alewife [20]. In Wisconsin, cisco and rainbow smelt co-occur in 17 lakes. Direct communication with fish managers in Wisconsin from six of the lakes with both cisco and rainbow smelt suggested that in three of those lakes, rainbow smelt invasion reduced or eliminated cisco recruitment and only larger cisco remained. In one of the lakes, cisco were extirpated following rainbow smelt invasion, and in 2 of those lakes, cisco did not experience any reductions. This suggests that the impact of rainbow smelt on cisco varies depending upon the physical and biological characteristics of the lake and importantly on how long the smelt have been present.

### Extirpation of cisco under rainbow smelt invasion and climate change scenarios

We found that rainbow smelt invasion has a smaller influence on cisco extirpations than does climate change. By the end of the century, cisco extirpations owing to climate change are expected to exceed extirpations driven by invasive rainbow smelt, unless one assumes invasion and impact rates that greatly exceed current estimates. Climate change plays a large role in species distributions and the modest increases in global air temperatures experienced between 1970 and 2000 has already altered the boundaries of the range of a panoply of species around the globe [64]. A comparison of multiple environmental stressors on amphibian declines in Italy similarly predicted that climate change leads to a greater loss of amphibian populations than direct habitat alteration or solar radiation [65]. However, variation in the impacts of different environmental stressors on freshwater ecosystems is expected among lakes owing to factors such as species interactions, community composition, and availability of refuge habitat [66].

The distribution and growth of rainbow smelt may also be altered by climate change. Rainbow smelt are a coldwater fish species preferring large, deep, clear, unproductive lakes and may experience similar responses as cisco populations to climate change [30]. As such, rainbow smelt may experience loss of habitat under climate change scenarios further reducing the impact of rainbow smelt on cisco populations in Wisconsin. Differential responses to climate change may be exhibited by different age classes of rainbow smelt. For example, in South Bay, Lake Huron, in years with shallower thermoclines, growth of age-1 rainbow smelt was reduced and growth of age-2 rainbow smelt was enhanced [53]. The differential response by age class could be attributed to two factors: i] adaptation of younger rainbow smelt to warmer conditions relative to older smelt [30], and ii] diel migration with strong dependence on thermocline depth which may alter the availability of food, feeding patterns, and distribution of rainbow smelt within a lake [53]. Our conceptual framework allows interactions among stressors. In this example, warming would be expected to make lakes less suitable for rainbow smelt. We were not able to explicitly model this since the original model forecasting smelt distribution did not include a climate variable. Declining suitability to rainbow smelt would further reduce the relative importance of smelt invasions relative to that of climate change and it would not likely alter the major conclusion that climate change trumps smelt in the extirpation of cisco in Wisconsin waters.

On a cautionary note, only models developed on biologically relevant variables based on biologically interpretable mechanisms that perform with high predictive ability under current scenarios should be extrapolated using climate change scenarios [9]. As such, the development of a framework to model environmental stressors in our example was limited only to the independent impacts of climate change and rainbow smelt invasions on cisco populations. However as our understanding of the broad-ranging impacts of climate change on lakes continues to increase, the probabilistic framework introduced here could be used to quantify the impacts of climate change and biological invasions on cisco populations in conjunction with other environmental stressors.

### Management implications

Our results highlight the threats to coldwater fish species. The probability of cisco extirpations could be reduced in Wisconsin through three interventions. First, the mitigation of climate change through the reduction of greenhouse gas emissions could significantly reduce the worst case losses of cisco. The population density function analyses in this study revealed a strong relationship between increases in mean annual air temperature and extirpation of cisco. As such, slowing the rate of warming could greatly reduce the potential magnitude of cisco extirpations across Wisconsin. Second, the dispersal of rainbow smelt into Wisconsin lakes is the result of natural and human factors [37], [62]. Regulations and public education can help reduce introductions of rainbow smelt, and reduce the probability of cisco extirpations. Third, as cisco require lakes with cold, well-oxygenated water, lake watershed protection efforts can be integrated into management efforts to enhance water quality in lakes by decreasing nutrient loads and increasing hypolimnetic oxygen concentrations [45]–[46]. However, should management efforts with respect to climate, biological invasions, and water quality not be incorporated into management strategies by identifying vulnerable lakes and implementing protection strategies, many cisco populations in this portion of their range are likely to be locally extirpated by 2100.

## Supporting Information

Table S1 **Summary of climate change models and scenarios used in the study.** Model name, country of origin, scenario, future time interval, increase in mean annual air temperature (°C) relative to current baseline (1961-1990) in Wisconsin, and predicted percent cisco loss in Wisconsin lakes.
(DOC)
