# Supplementary material for: Comparing Climate Change and Species Invasions as Drivers of Coldwater Fish Population Extirpations
Source: PLoS One. 2011 Aug 10;6(8):e22906. doi: 10.1371/journal.pone.0022906 (PMC3157906; doi:10.1371/journal.pone.0022906)
Supplement: Table S1 — Summary of climate change models and scenarios used in the study. Model name, country of origin, scenario, future time interval, increase in mean annual air temperature (°C) relative to current baseline (1961-1990) in Wisconsin, and predicted percent cisco loss in Wisconsin lakes. (DOC) [file pone.0022906.s001.doc]

**Table S1.** Summary of climate change models and scenarios used in the study. Model name, country of origin, scenario, future time interval, increase in mean annual air temperature (°C) relative to current baseline (1961-1990) in Wisconsin, and predicted percent cisco loss in Wisconsin lakes.

| **Model** | **Country of origin** | **Scenario** | **Year** | **Increase in MAT(°C)** | **Percent cisco loss** |
| --- | --- | --- | --- | --- | --- |
| CCCMA - CGCM3 | Canada | A1 | 2046-2065 | 3.48 | 38.24 |
| CCCMA - CGCM3 | Canada | A1 | 2081-2100 | 4.30 | 41.76 |
| CCCMA - CGCM3 | Canada | A2 | 2046-2065 | 3.20 | 35.88 |
| CCCMA - CGCM3 | Canada | A2 | 2081-2100 | 5.66 | 55.29 |
| CCCMA - CGCM3 | Canada | B1 | 2046-2065 | 2.46 | 31.76 |
| CCCMA - CGCM3 | Canada | B1 | 2081-2100 | 3.36 | 38.24 |
| CCCMA - CGCM3 - T63 | Canada | A1 | 2046-2065 | 3.91 | 39.41 |
| CCCMA - CGCM3 - T63 | Canada | A1 | 2081-2100 | 5.15 | 50.00 |
| CCCMA - CGCM3 - T63 | Canada | B1 | 2046-2065 | 3.12 | 34.12 |
| CCCMA - CGCM3 - T63 | Canada | B1 | 2081-2100 | 3.87 | 39.41 |
| CNRM-CM3 | France | A1 | 2046-2065 | 3.33 | 38.24 |
| CNRM-CM3 | France | A1 | 2081-2100 | 5.41 | 51.76 |
| CNRM-CM3 | France | A2 | 2046-2065 | 3.23 | 35.88 |
| CNRM-CM3 | France | A2 | 2081-2100 | 6.56 | 58.24 |
| CNRM-CM3 | France | B1 | 2046-2065 | 2.47 | 32.35 |
| CNRM-CM3 | France | B1 | 2081-2100 | 3.34 | 37.65 |
| CSIRO - MK3-0 | Australia | A1 | 2046-2065 | 1.94 | 28.82 |
| CSIRO - MK3-0 | Australia | A1 | 2081-2100 | 3.28 | 35.88 |
| CSIRO - MK3-0 | Australia | A2 | 2046-2065 | 2.57 | 32.35 |
| CSIRO - MK3-0 | Australia | A2 | 2081-2100 | 4.36 | 41.76 |
| CSIRO - MK3-0 | Australia | B1 | 2046-2065 | 1.27 | 25.88 |
| CSIRO - MK3-0 | Australia | B1 | 2081-2100 | 2.13 | 30.59 |
| CSIRO - MK3-5 | Australia | A1 | 2046-2065 | 3.40 | 38.24 |
| CSIRO - MK3-5 | Australia | A1 | 2081-2100 | 4.99 | 45.88 |
| CSIRO - MK3-5 | Australia | A2 | 2046-2065 | 3.12 | 34.12 |
| CSIRO - MK3-5 | Australia | A2 | 2081-2100 | 6.04 | 55.88 |
| CSIRO - MK3-5 | Australia | B1 | 2046-2065 | 2.47 | 31.76 |
| CSIRO - MK3-5 | Australia | B1 | 2081-2100 | 3.62 | 38.24 |
| GFDL - CM2 | United States (NOAA) | A1 | 2046-2065 | 3.35 | 38.24 |
| GFDL - CM2 | United States (NOAA) | A1 | 2081-2100 | 4.14 | 40.59 |
| GFDL - CM2 | United States (NOAA) | A2 | 2046-2065 | 2.90 | 34.12 |
| GFDL - CM2 | United States (NOAA) | A2 | 2081-2100 | 5.30 | 50.00 |
| GFDL - CM2 | United States (NOAA) | B1 | 2046-2065 | 2.39 | 31.18 |
| GFDL - CM2 | United States (NOAA) | B1 | 2081-2100 | 2.40 | 31.18 |
| GISS - AOM | United States (NASA) | A1 | 2046-2065 | 3.02 | 34.12 |
| GISS - AOM | United States (NASA) | A1 | 2081-2100 | 4.13 | 41.18 |
| GISS - AOM | United States (NASA) | B1 | 2046-2065 | 2.84 | 34.12 |
| GISS - AOM | United States (NASA) | B1 | 2081-2100 | 3.33 | 38.24 |
| IAP - FGOALS1 | China | A1 | 2046-2065 | 2.38 | 31.18 |
| IAP - FGOALS1 | China | A1 | 2081-2100 | 3.50 | 38.24 |
| IAP - FGOALS1 | China | B1 | 2046-2065 | 1.42 | 26.47 |
| IAP - FGOALS1 | China | B1 | 2081-2100 | 2.12 | 30.59 |
| INGV - ECHAM4 | Italy | A1 | 2046-2065 | 3.55 | 38.24 |
| INGV - ECHAM4 | Italy | A1 | 2081-2100 | 4.97 | 47.06 |
| INGV - ECHAM4 | Italy | A2 | 2046-2065 | 3.59 | 38.24 |
| INGV - ECHAM4 | Italy | A2 | 2081-2100 | 5.44 | 51.76 |
| IPSL - CM4 | France | A1 | 2046-2065 | 4.76 | 45.29 |
| IPSL - CM4 | France | A1 | 2081-2100 | 6.45 | 57.65 |
| IPSL - CM4 | France | A2 | 2046-2065 | 4.07 | 41.18 |
| IPSL - CM4 | France | A2 | 2081-2100 | 7.81 | 67.65 |
| IPSL - CM4 | France | B1 | 2046-2065 | 3.32 | 38.24 |
| IPSL - CM4 | France | B1 | 2081-2100 | 4.75 | 45.29 |
| MIROC3 - HIRES | Japan | A1 | 2046-2065 | 6.21 | 56.47 |
| MIROC3 - HIRES | Japan | A1 | 2081-2100 | 8.85 | 71.18 |
| MIROC3 - HIRES | Japan | B1 | 2046-2065 | 4.99 | 47.65 |
| MIROC3 - HIRES | Japan | B1 | 2081-2100 | 6.48 | 57.65 |
| MIROC3 - MEDRES | Japan | A1 | 2046-2065 | 4.80 | 46.47 |
| MIROC3 - MEDRES | Japan | A1 | 2081-2100 | 7.61 | 65.88 |
| MIROC3 - MEDRES | Japan | A2 | 2046-2065 | 4.60 | 44.71 |
| MIROC3 - MEDRES | Japan | A2 | 2081-2100 | 8.83 | 70.59 |
| MIROC3 - MEDRES | Japan | B1 | 2046-2065 | 3.66 | 38.24 |
| MIROC3 - MEDRES | Japan | B1 | 2081-2100 | 4.95 | 47.65 |
| MIUB - ECHO | Germany | A1 | 2046-2065 | 4.25 | 41.76 |
| MIUB - ECHO | Germany | A1 | 2081-2100 | 6.64 | 58.82 |
| MIUB - ECHO | Germany | A2 | 2046-2065 | 4.62 | 44.71 |
| MIUB - ECHO | Germany | A2 | 2081-2100 | 6.95 | 61.18 |
| MIUB - ECHO | Germany | B1 | 2046-2065 | 3.27 | 37.06 |
| MIUB - ECHO | Germany | B1 | 2081-2100 | 4.98 | 47.65 |
| MPI - ECHAM5 | Germany | A2 | 2046-2065 | 2.37 | 31.18 |
| MPI - ECHAM5 | Germany | A2 | 2081-2100 | 4.85 | 47.06 |
| MPI - ECHAM5 | Germany | B1 | 2046-2065 | 2.02 | 29.41 |
| MPI - ECHAM5 | Germany | B1 | 2081-2100 | 3.40 | 38.24 |
| MRI - CGCM2 | Japan | A1 | 2046-2065 | 2.95 | 34.12 |
| MRI - CGCM2 | Japan | A1 | 2081-2100 | 3.99 | 39.41 |
| MRI - CGCM2 | Japan | A2 | 2046-2065 | 2.75 | 34.12 |
| MRI - CGCM2 | Japan | A2 | 2081-2100 | 4.73 | 45.88 |
| MRI - CGCM2 | Japan | B1 | 2046-2065 | 2.04 | 29.41 |
| MRI - CGCM2 | Japan | B1 | 2081-2100 | 3.33 | 37.65 |

Summary of climate change models and scenarios used in the study. Model name, country of origin, scenario, future time interval, increase in mean annual air temperature (°C) relative to current baseline (1961-1990) in Wisconsin, and predicted percent cisco loss in Wisconsin lakes.
